# A protocol for anti-CD4 IgG antibody purification using plasma samples from people with HIV and antibody-mediated cytotoxicity

**DOI:** 10.1016/j.mex.2024.102698

**Published:** 2024-04-15

**Authors:** Zhenwu Luo, Wei Jiang

**Affiliations:** a Department of Microbiology and Immunology, Medical University of South Carolina, Charleston, SC, USA; b Division of Infectious Diseases, Department of Medicine, Medical University of South Carolina, Charleston, SC, USA; c Ralph H. Johnson VA Medical Center, Charleston, SC, USA

**Keywords:** Antibody-mediated cytotoxicity (ADCC), Anti-CD4 IgG autoantibodies, Anti-CD4 IgG purification from plasma samples, Immune reconstitution, HIV, Antiretroviral therapy, Antibody purification and mediated cytotoxicity

## Abstract

Background. Up to 20% of people with HIV (PWH) fail to recover their CD4+ *T* cell counts to levels similar to healthy controls after suppressive antiretroviral therapy (ART). Immune non-responders (INRs) are PWH on suppressive ART with CD4+ *T* cell counts lower than 350 cells/mL, whereas their CD8+ *T* cell counts are higher than healthy controls. We are the first group to report that increased anti-CD4 autoantibody IgGs in INRs are responsible for blunted CD4+ *T* cell reconstitution in PWH with ART and viral suppression through anti-CD4 IgG-induced antibody-mediated cytotoxicity (ADCC) against CD4+ *T* cells *in vitro*. Notably, anti-CD4 IgG-mediated poor CD4+ *T* cell recovery from suppressive ART is the only mechanism targeting CD4+ *T* cells, specifically. Results. We provide a detailed one-by-one step protocol from antigen-specific antibody isolation using plasma samples, to ADCC assay. Conclusions. To promote reproducible research, a detailed protocol for isolating anti-CD4 IgG autoantibodies from plasma samples of PWH and evaluating ADCC effects is reported here.

• Antigen-specific antibody isolation using human plasma samples
• Antibody-mediated cytotoxicity (ADCC)

## Graphical abstract

Anti-CD4 autoantibody IgGs in INRs bind to CD4 on the surface of CD4+ *T* cells through the antigen-binding region and also bind to FcR on NK cells, which triggers antibody-mediated NK cytotoxicity and CD4+ *T* cell death. This antibody-mediated cytotoxicity (ADCC) against CD4+ *T* cells may be responsible for blunted CD4+ *T* cell reconstitution in PWH with ART and viral suppression.

**Figure:**
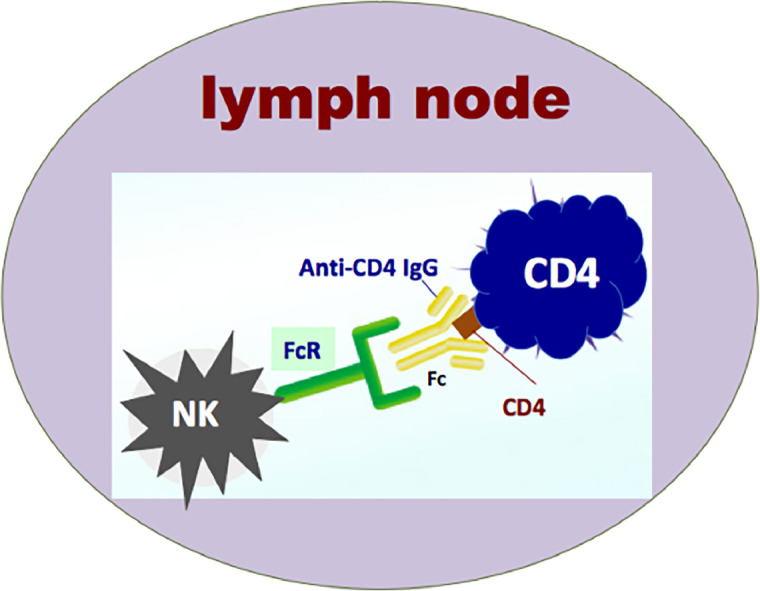

Specifications table

**Table:** 

Subject area:	Immunology and Microbiology
More specific subject area:	Antibody-mediated cytotoxicity
Name of your method:	Antibody purification and mediated cytotoxicity
Name and reference of original method:	NA
Resource availability:	NA

## Method details

After long-term antiretroviral therapy (ART) and peripheral viral suppression, up to 20% of people with HIV (PWH) fail to recover their CD4+ *T* cell counts to levels similar to healthy controls. CD4+ *T* cell decline characterizes these immune non-responders (INRs, aviremic/ART+, CD4+ *T* cell counts < 350 cells/mL); moreover, their CD8+ *T* cell counts are higher than healthy controls (1). We are the first group to report that plasma levels of anti-CD4 autoantibody IgGs are increased in INRs compared to immune responders (IRs) and uninfected controls, and purified anti-CD4 IgGs from plasma samples of INRs induced CD4+ *T* cell death through antibody-mediated cytotoxicity (ADCC) *in vitro* (1–7). Notably, anti-CD4 IgG-mediated poor CD4+ *T* cell recovery from suppressive ART is the only mechanism targeting CD4+ *T* cells, specifically. Other known mechanisms (e.g., thymic and lymph node fibrosis, chronic immune activation, leaky gut, and increased microbial translocation) may not account for decreased CD4+ *T* cell counts specifically in INRs.

Recently, there are studies and interests related to autoimmunity and anti-CD4 autoantibodies in HIV, though there is a debate about whether anti-CD4 autoantibodies mediate CD4+ *T* cell death through antibody-mediated cytotoxicity (8), which has conflicting results of our previous studies (1, 3, 9, 10)mainly due to the challenging technologies (antigen-specific antibody isolation from plasma and ADCC). To increase transparency and research reproducibility, we show methodology and explain the details of key steps in the anti-CD4 autoantibody isolation from plasma samples and the ADCC assay to allow reproducibility in the future. This protocol for isolating anti-CD4 antibodies also can be used to isolate any antigen-specific antibodies from plasma or from antibody containing fluids, using the desired antigens.

## Step 1 buffer exchange

➢ Amicon Ultra 0.5 mL filters (10 kDa, Millipore, Catalog number: UFC5010)
  ➢ 1X PBS
  ➢ Recombinant soluble CD4 protein (Recombinant Soluble CD4, amino acids 1–370, produced in mammalian CHO cells and purity > 95% by SDS-PAGE, Progenics Pharmaceuticals, Inc, New York)

1. Insert an Amicon® Ultra-0.5 device into one microcentrifuge tube provided in the kit (Fig. 1).

**Fig. 1:**
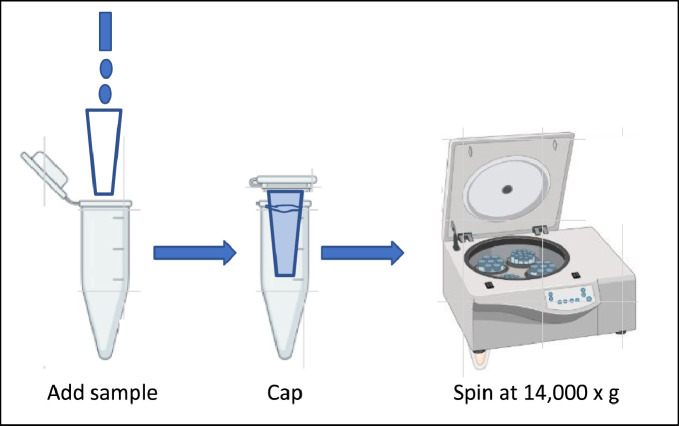
Target protein uploading to Amicon Ultra filters. Amicon Ultra 0.5 mL filters (10 kDa, Millipore, Catalog number: UFC5010).
2. Add 50 µL (1 mg/mL) sCD4 to the Amicon® Ultra filter device.
3. Add 400 µL PBS and cap it, spin the device at 14,000 × g for approximately 7 min (Fig. 1).
4. Repeat step 3 two times.
5. To recover the concentrated solute, place the Amicon® Ultra filter device upside down in a clean microcentrifuge tube. Spin for 2 min at 1000 × *g* to transfer the concentrated sample from the device to the tube (Fig. 2).

**Fig. 2:**
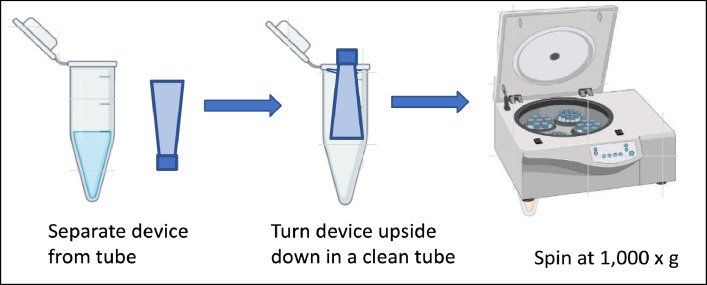
Transfer the concentrated sample from the device to the tube.

## Step 2 recombinant soluble CD4 protein binding to the magnetic bead

➢ NHS Mag Sepharose (GE Healthcare, Catalog number**:** 28-9440-09)
➢ NHS HP SpinTrap Buffer Kit (GE Healthcare, Catalog number: 28-9135-69)
➢ Magnetic rack (Cytiva, MagRack 6, Catalog number: 28948964)

**Note:** All steps are strictly according to the handbook in the NHS Mag Sepharose kit:

➢ Add the protein from step 1 to a 1.5 mL Eppendorf tube, then add the coupling buffer from the kit, and keep it on ice.
➢ Prepare 1 mM HCl and make sure it is ice-cold before use.

1. Magnetic bead preparation
  A. Dispense the 100uL magnetic beads into a 1.5 mL Eppendorf tube.
  B. Place the Eppendorf tube in the magnetic rack and remove the storage solution.
2. Equilibration
  A. Add 500 µL ice cold equilibration buffer.
  B. Resuspend the medium.
  C. Remove the liquid.
3. Binding of CD4 protein
  A. After equilibration, add CD4 protein solution immediately.
  B. Resuspend the medium and incubate with slow end-over-end mixing (slowly rotate at 500 rpm on a rotator to prevent beads precipitation) for 60 min.
  C. Remove the liquid.
4. Blocking of residual active groups
  A. Add 500 µL blocking buffer A and remove the liquid.
  B. Add 500 µL blocking buffer B and remove the liquid.
  C. Add 500 µL blocking buffer A.
  D. Incubate for 15 min with slow end-over-end mixing.
  E. Remove the liquid.
  F. Add 500 µL blocking buffer B and remove the liquid.
  G. Add 500 µL blocking buffer A and remove the liquid.
  H. Add 500 µL blocking buffer B and remove the liquid.
5. Equilibration for binding
  A. Add 500 µL binding buffer.
  B. Remove the liquid.

## Step 3 purify the isolated anti-CD4 antibodies from plasma

1. Binding of the target protein
  A. Add 100 uL labeled beads to a 50 mL tube, add 20 mL plasma containing the anti-CD4 antibodies to the 50 mL tube. Add 20 mL wash/bind buffer containing 2 M Urea.
  B. Incubate with slow end-over-end mixing (slowly rotate at 200 rpm on a rotator to prevent beads precipitation) for 4 h at 4 °C or room temperature for 2 h.
  C. Briefly spin at 10 g for 1 min, remove the supernatant (containing non-bound antibodies, which may be stored for future usage) and collect pellets.
2. Wash (perform this step 3 times totally)
  A. Add 500 µL wash buffer, mix, then place on the magnetic rack.
  B. Remove the liquid.
3. Elution (perform this step 2 times totally)
  A. Add 500 uL volumes of elution buffer for each the 50 µL magnetic bead volume.
  B. Fully resuspend the medium and incubate for at least 2 min.
  C. Remove and collect the elution fraction.

**Note:** Elution should contain 2 M Urea (VWR, Catalog number: 17-1319-01).

## Step 4 exchange buffer and concentrate protein

1. Insert an Amicon® Ultra-0.5 device into one microcentrifuge tube provided in the kit.
2. Add the 500 uL purified anti-CD4 antibodies to an Amicon® Ultra filter device.
3. Spin the device at 14,000 × *g* for approximately 5 min. Decant the liquid that flowed through the filter.
4. Repeat steps 2-3 until all purified anti-CD4 antibodies are concentrated.
5. Add 400 uL PBS, spin the device at 14,000 × g for approximately 5 min. Decant the liquid that flowed through the filter.
6. Repeat step 5 two times.
7. To recover the concentrated solute, place the Amicon® Ultra filter device upside down in a clean microcentrifuge tube. Spin for 2 min at 1000 × g to transfer the concentrated sample from the device to the tube.

## Step 5 isolation of anti-CD4 IgGs

The isolated anti-CD4 antibodies contain all classes of antibodies (e.g., IgA, IgM, IgG). Since IgG is the main class of antibodies presenting with ADCC activity, next we will isolate IgG from the anti-CD4 antibodies.

➢ Protein A IgG purification kit (Catalog number: 44667, ThermoScientific)
➢ Amicon Ultra-0.5 Centrifugal Filter Unit (Catolog number: UFC503096, Millipore)

1. Remove the end write cover from the column.
2. Insert the column into a 15 ml tube.
3. Add 1 mL binding buffer to wash the column, perform 3 times, do not let the column dry completely.
4. When column is almost dry, add 200–500ul plasma; let IgG bind to the column
5. Add 1 mL binding buffer to wash the unbounded antibodies, perform 5 times.
6. Add 1 mL elution buffer to wash the column; because the first wash buffer has the most IgG, the second wash can occur with 0.5 ml elution buffer.
7. Add nanodrop to calculate protein concentration; wash nanodrop, add 2uL elution buffer to adjust for blank; add sample 2uL to measure protein concentration in the sample.
8. To reuse the column, release all binding IgGs; add 1 mL elution buffer, perform 3 times to release the binding IgGs. Then add 1 mL binding buffer, l wash 3 times, leave 1 ml binding buffer; cap and store for future use.
9. Next to concentrate IgGs and change buffer to PBS since elution buffer could impair IgGs.
  9a. Use Amicon Ultra-0.5 ml centrifugal filters 30k (IgG is about 150k)
  9b. Add the filter into a 2 ml vial provided in the kit
  9c. Add 0.5 mL isolated IgGs in elution buffer from step 8 to the column, then 14,000 g 10 min, repeat until all elution buffer uses.
  9d. Add PBS 0.5 ml, 14,000 g 10 min, 3 times
  9e. Reverse the column in a new tube, then centrifuge 1000 g for 2 min. This produces the concentrated IgGs, then add PBS to 0.5–1 mg/mL, aliquot and store at −80C to avoid repeated freezing and thawing.

## Step 6 ADCC

1. Target cells (CD4+ *T* cells) and Effect cells (NK cells) obtained from non-immune responder HIV patients (undetectable plasma viral load, CD4+ *T* cell counts < 350 cells/µL) on antiretroviral therapy.
  Use the enrichment kit, strictly following the protocol provided in the kit.
  • EasySep™ Human CD4 Cell Enrichment Kit (STEMCELL, Catalog number: 19052)
  • EasySep™ Human NK Cell Enrichment Kit (STEMCELL, Catalog number: 19055)

This procedure is used for processing 250 µL – 2 mL of sample (up to 1 × 10^8^ cells).

A. Prepare a mononuclear cell suspension at a concentration of 5 × 10^7^ cells/mL in the Separation Buffer. Cells were placed in a 4.5 or 5 mL polystyrene tube to properly fit into the Purple EasySep™ Magnet.
  a. Separation buffer: PBS PH7.2, supplemented with 0.5%BSA and 2 mM EDTA
  b. Falcon™ 5 mL Polystyrene Round-Bottom Tubes (BD Biosciences, Catalog number: 352058) or 4.5 mL polystyrene tubes (Catalog number: 120161, 4.5 mL PS tube, Greiner bio-one) are recommended.
B. Add the EasySep™ Human CD4 or NK Cell Enrichment Cocktail at 50 µL/mL cells (e.g. for 2 mL of cells, add 100 µL of cocktail). Mix well and incubate at room temperature (15–25 °C) for 10 min.
C. Vortex the EasySep™ D Magnetic Particles for 30 s. Ensure that the particles are in a uniform suspension with no visible aggregates.
D. Add the EasySep™ D Magnetic Particles at 100 µL/mL cells (e.g. for 2 mL of cells, add 200 µL of magnetic particles). Mix well and incubate at room temperature (15–25 °C) for 5 min.
E. Bring the cell suspension up to a total volume of 2.5 mL by adding separation buffer. Mix the cells in the tube by gently pipetting up and down 2 - 3 times. Place the tube (without cap) into the magnet. Set aside for 5 min.
F. Pick up the EasySep™ Magnet, and in one continuous motion invert the magnet and tube, pouring off the desired fraction into a new 4.5 mL polystyrene tube. The magnetically labeled unwanted cells will remain bound inside the original tube, held by the magnetic field of the EasySep™ Magnet. Leave the magnet and tube in an inverted position for 2-3 s, then return to upright position.
  a. **Do not shake or blot off any drops that may remain hanging from the mouth of the tube.**
  b. **From the following step, the separated NK cells should be kept at 4** °**C, until the NK cells have been added in the 96 wells plate.**
G. Wash the cells with **pre-cold separation buffer** 2 times; the negatively selected, enriched cells in the new tube in R10 are now ready for use.

R10: RPMI+10% FBS

➢ Use the R10 buffer in all these steps, unless a different specified buffer is needed.
2. CD4**+ *T* cells depletion**
  A. Dilute the cell at 5 × 10^5^/mL in R10, add 50 µL to the 96 well V bottom plate, with CD4+ *T* cells 2.5 × 10^4^ per well.
    R10: RPMI+10% FBS
  B. Using the R10 at all of steps, unless the specific explain
  C. Add the anti-CD4 antibody (from 1 µg/mL, 2.5 µg/mL, to 5 µg/mL, you may need to titrate your isolated anti-CD4 IgGs from 1 to 10 µg/mL), and purified IgG or positive control (hIgG OKT3, 0.5 µg/mL) to the CD4+ *T* cells, culture the cells at 37 °C for 30 min.
  D. Dilute the NK cells at 1.5 × 106/mL in R10, add 50 µL to the 96 well V bottom plate, NK cells 7.5 × 104 per well, NK cells: CD4+ *T* cells at a ratio of 3:1, with each well containing 1 × 105 total cells per well.
  E. Culture the cells at room temperature for 15 min, then spin the plate at 300 g for 1 min.
  F. Incubate plates for 6 h at 37 °C.
  G. Stain the cells with CD4 and CD3, then Annexin V in annexin V buffer (BD, Catalog number: 556454)
  H. Run on flow cytometry within 30 min.
3. Antibody **dependent NK activation.**
  A. Dilute the cells at 5 × 10^5^/mL in R10, add 50 µL to the 96 well V bottom plate, with CD4+ *T* cells 2.5 × 10^4^ per well.
    R10: RPMI+10% FBS
  B. Add the anti-CD4 antibody and purified IgG or positive control (hIgG OKT3) to the CD4+*T* cells; culture the cells at 37 °C for 15–30 min.
  C. Dilute the NK cells at 5 × 105/mL in R10, add 50 µL to the 96 well U bottom plate, NK cells 2.5 × 104 per well, NK cells: CD4+ *T* cells at 1:1, with each well containing 5 × 104 total cells.
  D. Add the CD107a-BV 510 antibody (Catalog number: 563078, BD) 2 µL/well, the GolgiStop™ Protein Transport Inhibitor (brefeldin A, Catalog number: 554724, BD) and GolgiPlug™ Protein Transport Inhibitor (monensin, Catalog number: 555029, BD) at the final concentration of 1 µL/mL.
  E. Culture the cells at room temperature for 15 min, then spin the plate at 250 g for 4 min.
  F. Incubate plates for 6 h at 37 °C.
  G. Stain the cells with Ghost Red 780 and CD3.
  H. Add 500 µL 1x PBS to wash the cells, 350 g, 5 min, decant the supernatant. Vortex the cells for 5 s.
  I. Add 200 µL BD cytofix/cytoperm (BD, 51-2090KZ) 200 µL per tube, 4 °C for 20 min. Concurrently, prepare the 1X BD perm/Wash buffer (BD, catalog number: 51-2091KZ).
  J. Add 300 µL 1X BD perm/Wash buffer to each tube, 450 g, 5 min, decant the supernatant.
  K. Add 2 µL IFN-r antibody for intracellular staining, 4 °C, 30 min, avoid light.
  L. Add 500 µL 1X BD perm/Wash buffer to each tube, 450 g, 5 min, decant the supernatant.
  M. Wash again.
  N. Add 100–150 µL 2–4%PFA (Biolegend, Catalog number: 420801, 4% PFA) to each tube. Refrigerate the tube in 4 °C to prepare to run the samples using flow cytometry.

## Ethics statements

This is a non-human subject study and no ethics approval is involved.

## CRediT authorship contribution statement

**Zhenwu Luo:** Validation, Writing – original draft, Methodology, Conceptualization. **Wei Jiang:** Writing – review & editing, Supervision, Conceptualization, Project administration, Funding acquisition.

## Declaration of competing interest

The authors declare that they have no known competing financial interests or personal relationships that could have appeared to influence the work reported in this paper.

## Data Availability

No data was used for the research described in the article.

